# Network toxicology and machine learning reveal the toxicological impact of Bisphenol A exposure on osteoarthritis

**DOI:** 10.1097/MD.0000000000045406

**Published:** 2025-10-31

**Authors:** Jing Tan, Daobin Han

**Affiliations:** a Department of Rehabilitation Medicine, Central Hospital of Guangdong Provincial Nongken, Zhanjiang, Guangdong, China; b Medical Laboratory Center, Qilu Second Hospital of Shandong University, Jinan, Shandong, China.

**Keywords:** Bisphenol A, machine learning, molecular docking, network toxicology, osteoarthritis, SHAP

## Abstract

Osteoarthritis (OA) is the most common chronic degenerative joint disease, and increasing evidence suggests that environmental exposures play a crucial role in its onset and progression. Bisphenol A (BPA), a widespread environmental endocrine disruptor, has been associated with inflammation, oxidative stress, and abnormal bone metabolism; however, its potential mechanistic involvement in OA development remains unclear. In this study, BPA targets were 1st predicted using the CHEMBL, SwissTargetPrediction, and Similarity Ensemble Approach databases. Eight OA transcriptomic datasets were retrieved and integrated from the Gene Expression Omnibus database to construct training and validation cohorts. Differential expression analysis and weighted gene co-expression network analysis were performed on the training cohort, and their union was used to identify OA-related key genes. BPA targets were intersected with OA key genes to establish a BPA–OA interaction network using Cytoscape, followed by functional enrichment analyses. Based on the intersecting genes, 113 machine learning models were applied to identify the optimal predictive model, and SHapley Additive exPlanations analysis was conducted to interpret feature contributions. Core genes were subsequently identified and validated through molecular docking to assess their binding stability with BPA. A total of 235 potential BPA targets were predicted across the 3 databases. From the Gene Expression Omnibus database, 8 datasets were obtained, of which 5 were integrated as the training set and 3 as the validation set. In the training set, 541 differentially expressed genes were identified, and weighted gene co-expression network analysis yielded 3575 genes; their union resulted in 3739 OA-related genes. The intersection of these genes with BPA targets produced 47 candidate genes. Functional enrichment analysis revealed significant involvement in cytokine signaling, calcium signaling, lipid metabolism, and TRP channel-related pathways. Among the 113 machine learning models, the plsRglm ensemble model performed best (mean area under the receiver operating characteristic curve = 0.901). SHapley Additive exPlanations analysis further identified CLK1, PTPRC, and ALDH5A1 as core targets. Molecular docking confirmed stable binding of BPA to these proteins, with binding energies below −5 kcal/mol. This study systematically elucidates, for the 1st time, the potential mechanisms by which BPA may contribute to OA progression via inflammation- and oxidative stress-related pathways, using a network toxicology and machine learning framework. Furthermore, CLK1, PTPRC, and ALDH5A1 were identified as key targets, providing novel insights into the environmental toxicology of OA and potential therapeutic targets.

## 1. Introduction

Osteoarthritis (OA) is a prevalent chronic degenerative joint disease characterized by progressive cartilage degradation, synovial inflammation, osteophyte formation, and joint space narrowing.^[1]^ Traditionally, OA has been attributed primarily to mechanical loading and aging; however, recent studies increasingly highlight the significant role of environmental factors in its onset and progression. For instance, evidence suggests that the global prevalence of OA is closely linked to modern lifestyle-related factors such as obesity, poor diet, and physical inactivity, underscoring the need for preventive strategies from an environmental perspective.^[2]^ Moreover, a systematic review detected various persistent environmental pollutants (including lead and polychlorinated biphenyls) in human osteoarticular tissues, which may contribute to OA pathogenesis by inducing chondrocyte apoptosis and oxidative damage.^[3]^ Large-scale cohort studies further demonstrate a positive association between long-term exposure to air pollutants such as PM_10_ and NO_2_ and OA incidence.^[4]^ Despite these observations, the precise molecular mechanisms linking environmental pollutants to OA pathophysiology remain insufficiently explored, representing an important research gap.

Bisphenol A (BPA) is a ubiquitous industrial chemical widely used in the production of polycarbonate plastics and epoxy resins, and it serves as a raw material for various household products, including electronic devices, children’s toys, kitchenware, water pipes, and reusable bottles. BPA is recognized as a typical environmental endocrine disruptor.^[5,6]^ Humans are exposed to BPA both directly through ingestion and dermal contact, and indirectly via environmental contamination and the food chain.^[7–10]^ BPA exerts estrogenic activity by binding to estrogen receptors^[11]^ and displays anti-androgenic effects by enhancing aromatase activity, which converts androgens into estrogens.^[12,13]^ Additionally, BPA stimulates the production of pro-inflammatory cytokines (e.g., TNF-α, IL-6) to elicit inflammatory responses,^[14]^ while also inducing oxidative stress through mitochondrial dysfunction, downregulation of antioxidant enzymes, and disruption of intracellular signaling pathways.^[15–17]^ Both inflammation and oxidative stress are closely associated with impaired bone health, implying that BPA may exert degenerative effects on the skeletal system.^[18–20]^ However, only limited studies have systematically investigated whether BPA directly contributes to OA development through molecular mechanisms, leaving a critical knowledge gap.

**Figure 1.**
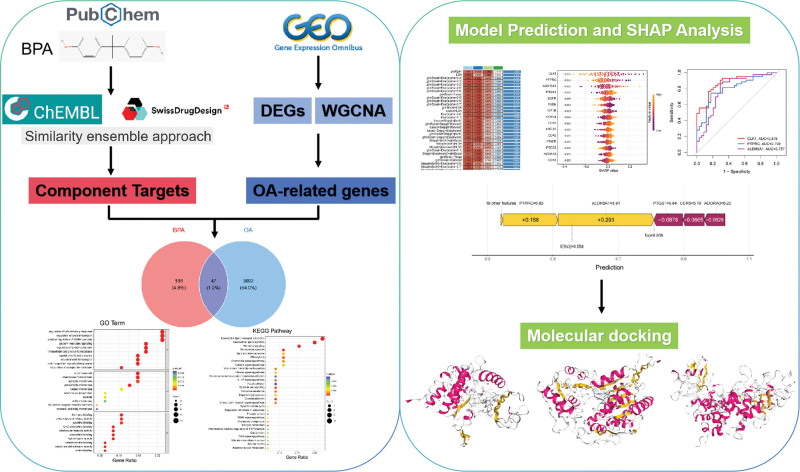
Flowchart of dataset analysis in this paper.

Therefore, elucidating the link between BPA exposure and OA, along with identifying its molecular targets, is of great importance for clarifying the environmental contribution to OA and discovering novel therapeutic targets. To address this gap, this study integrated network toxicology and transcriptomic analyses with machine learning and molecular docking techniques to systematically explore the molecular connections between BPA and OA from an environmental exposure perspective. As illustrated in Figure 1, BPA targets were predicted and cross-analyzed with OA-related key genes to identify shared targets, followed by machine learning and SHapley Additive exPlanations (SHAP)-based feature interpretation to screen core genes. Finally, molecular docking was performed to validate the binding characteristics of BPA with these targets. This work aims to provide novel mechanistic insights into environmentally driven OA and to establish a theoretical basis for future interventions.

## 2. Materials and methods

### 2.1. Identification of BPA targets

BPA was 1st retrieved from the PubChem database to obtain its 2D structure, 3D structure, and SMILES format. Subsequently, its potential targets were predicted using the following 3 databases:

- CHEMBL (https://www.ebi.ac.uk/chembl/): “Bisphenol A” was used as the search term, with the species restricted to “Human,” and its known targets were extracted to form the 1st target set.
- SwissTargetPrediction (http://www.swisstargetprediction.ch/): The SMILES structure of BPA was uploaded to predict potential targets, generating the 2nd target set.
- Similarity Ensemble Approach (https://sea.bkslab.org/): The SMILES structure was similarly used to predict additional potential targets, forming the 3rd target set.

To standardize target naming and annotation, all obtained proteins were normalized using the UniProt database. Finally, the ggvenn R package (R Foundation for Statistical Computing, Vienna, Austria, version, R 4.4.1) was employed to merge the 3 predicted target sets and derive the final list of BPA potential targets.

### 2.2. Identification of OA targets

#### 2.2.1. Acquisition and preprocessing of OA transcriptomic datasets

Eight transcriptomic microarray datasets containing samples from both healthy controls and patients with OA were downloaded from the NCBI Gene Expression Omnibus (GEO) database. First, background correction and normalization were performed for each dataset using the limma package. Subsequently, 5 datasets generated on the same sequencing platform were merged using the ComBat algorithm from the sva package to remove batch effects and were designated as the training cohort for primary analyses. The remaining 3 datasets served as an independent validation cohort for model evaluation.

To assess the effectiveness of batch effect correction, boxplots of gene expression levels and 2-dimensional principal component analysis plots were generated using the reshape2 and ggplot2 packages, respectively, to compare sample distributions before and after batch correction. The basic characteristics of the included datasets are summarized in Table 1.

**Table 1:** Information on the gene datasets.

Type	GEO ID	Platform documents	Normal group	Disease group	Tissue
Training set	GSE55235	GPL96	10	10	Synovium
	GSE55457	GPL96	10	10	Synovium
	GSE55584	GPL96	0	6	Synovium
	GSE82107	GPL570	7	10	Synovium
	GSE206848	GPL570	7	7	Synovium
Test set	GSE46750	GPL10558	12	12	Synovium
	GSE169077	GPL96	5	6	Cartilage
	GSE178557	GPL13497	4	4	Cartilage

GEO = Gene Expression Omnibus.

#### 2.2.2. Differential expression analysis and weighted gene co-expression network analysis (WGCNA)-based identification of OA-related genes

In the training cohort, differential expression analysis was performed using the limma package, with the Benjamini–Hochberg method applied for false discovery rate correction. Genes with adjusted *P* < .05 and |log2fold change| ≥ 0.585 were identified as differentially expressed genes (DEGs) between healthy and OA samples.

Subsequently, WGCNA was conducted to identify gene modules closely associated with the OA phenotype. Genes with a standard deviation >0.5 were selected for network construction using the WGCNA package. The goodSamplesGenes function was applied to filter out genes or samples with excessive missing values. The pickSoftThreshold function was used to determine an appropriate soft-thresholding power to ensure scale-free topology. The adjacency matrix was then transformed into a topological overlap matrix, and gene modules were initially identified using the cutreeDynamic function. Modules with similar eigengenes were subsequently merged based on module eigengene correlations. Pearson correlation coefficients were calculated between each module and the OA phenotype, and a module–trait heatmap was generated. The genes from the 2 modules showing the strongest correlations with OA were defined as WGCNA-derived OA-related module genes. Finally, the union of DEGs and WGCNA module genes was taken to derive the potential OA key genes.

### 2.3. Construction of BPA–OA target network and functional enrichment analysis

To identify potential targets through which BPA may modulate OA pathogenesis, the intersection of predicted BPA targets and OA key genes was determined using the ggvenn R package. The resulting intersecting genes were visualized using a regulatory interaction network constructed in Cytoscape. Gene Ontology and Kyoto Encyclopedia of Genes and Genomes pathway enrichment analyses were then performed for the intersecting genes using the clusterProfiler package, with significance thresholds set at adjusted *P* < .05.

### 2.4. Multi-model prediction and SHAP-based feature interpretation

To further identify core targets of BPA in OA pathogenesis, we established an integrated machine learning framework combining multiple algorithms. A total of 113 predictive models were constructed using 10 classical machine learning algorithms (Lasso, SVM, RF, glmBoost, Stepwise GLM, Ridge, Elastic Net, GBM, LDA, XGBoost, and Naive Bayes). Model performance was evaluated in both the training and validation cohorts, with the mean area under the receiver operating characteristic curve (AUC) used as the primary metric. Model performance was visualized via heatmaps, and the model with the highest mean AUC was selected as the optimal model.

To interpret feature contributions within the optimal model, we employed SHAP. The expression matrix of model-selected genes was extracted, and SHAP values were computed using the kernelshap R package to quantify each gene’s contribution to model prediction. Visualization was performed using the shapviz package, and genes with a mean absolute SHAP value >0.05 were defined as potential core factors involved in BPA-mediated OA regulation.

### 2.5. Molecular docking

To validate whether SHAP-identified core genes could serve as potential targets of BPA in OA, molecular docking analyses were performed. Protein structures corresponding to genes with mean absolute SHAP values > 0.05 were retrieved from the RCSB Protein Data Bank, prioritizing high-resolution, structurally complete models. The 3D structure of BPA was downloaded from PubChem and converted to PDB format for docking.

Molecular docking was conducted using AutoDock Vina to predict binding conformations and calculate binding energies. The binding pose with the lowest predicted binding energy was selected as the optimal conformation. Finally, docking complexes were visualized using PyMOL to assess the binding interactions.

## 3. Results

### 3.1. Toxicological information and target prediction of BPA

**Figure 2.**
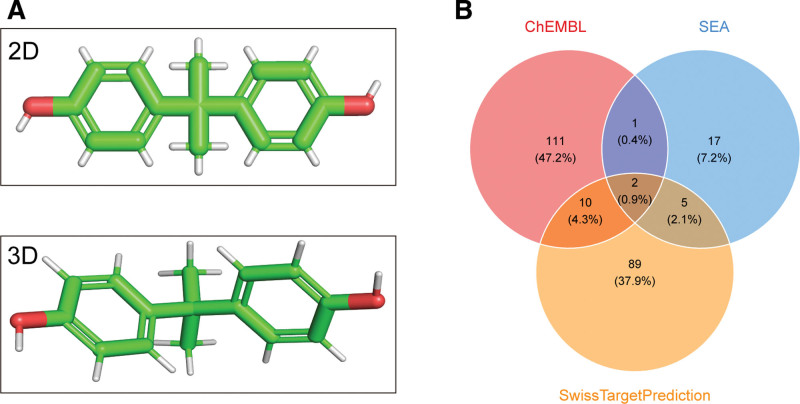
Toxicological information and target prediction of BPA. (A) 2D and 3D molecular structures of BPA; (B) Venn diagram of predicted targets from 3 databases. BPA = Bisphenol A.

Basic chemical information on BPA was retrieved from the PubChem database, and its 2D and 3D molecular structures are shown in Figure 2A. The SMILES representation of BPA is: CC(C) (C1 = CC = C(C = C1)O)C2 = CC = C(C = C2)O. Based on this structure, potential targets of BPA were predicted using 3 online databases: CHEMBL, SwissTargetPrediction, and Similarity Ensemble Approach, which identified 124, 100, and 25 targets, respectively (Table S1, Supplemental Digital Content, https://links.lww.com/MD/Q594). After merging the results, a total of 235 potential targets for BPA were obtained (Fig. 2B).

### 3.2. Identification of potential key genes in OA

#### 3.2.1. Processing of GEO datasets and differential expression analysis

**Figure 3.**
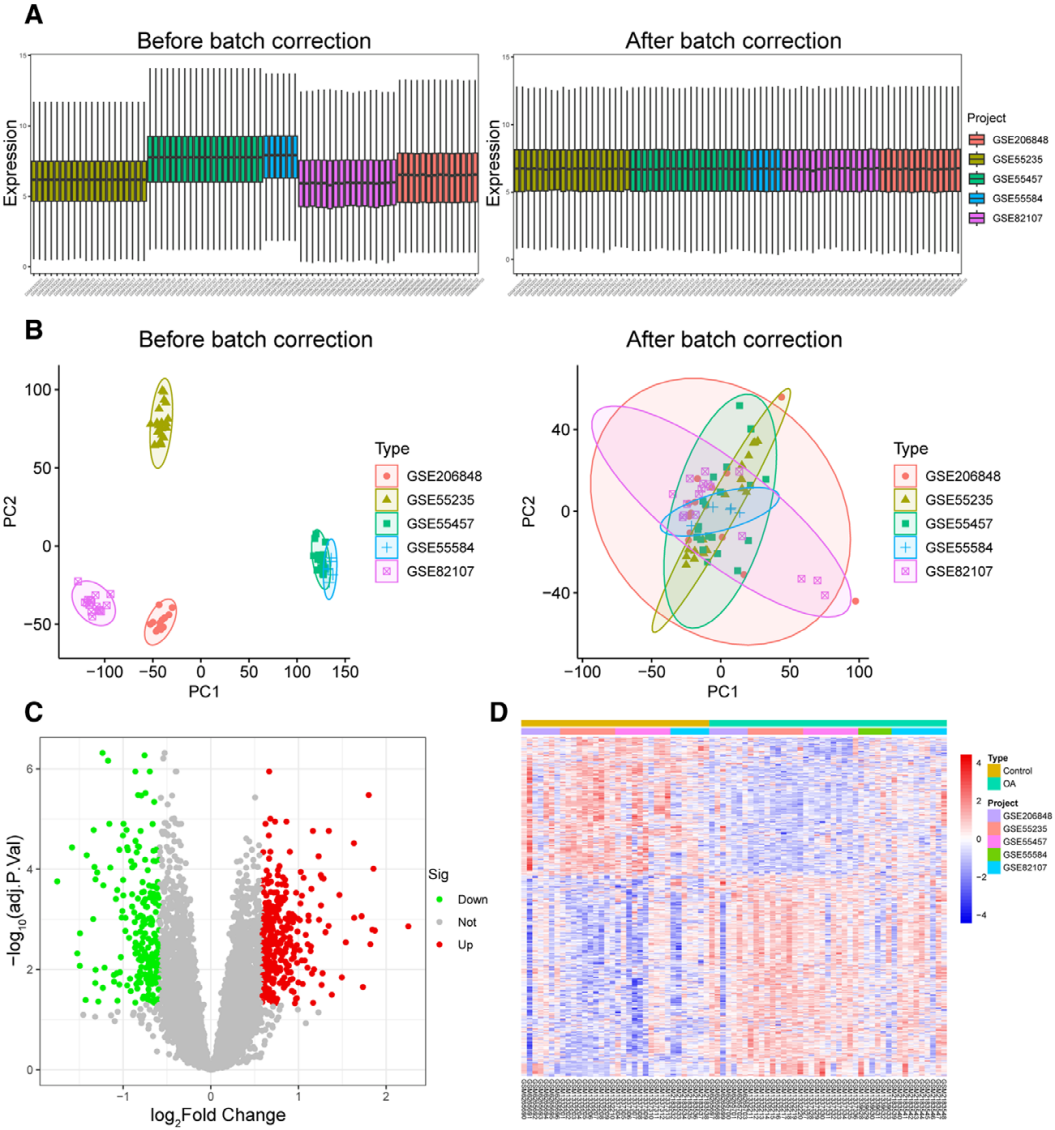
Processing of GEO datasets and differential expression analysis. (A) Boxplots of sample expression distributions before and after dataset merging; (B) PCA plots before and after batch effect correction; (C–D) Volcano plot and heatmap of differentially expressed genes. GEO = Gene Expression Omnibus, PCA = principal component analysis.

Five GEO datasets were integrated to form the training cohort (Table S2, Supplemental Digital Content, http://links.lww.com/MD/Q700). To minimize inter-dataset batch effects, normalization and batch correction were performed. The distribution of gene expression values before and after correction is shown in Figure 3A. Prior to correction, substantial differences in expression profiles were observed across datasets, whereas post-correction, sample expression levels became more uniform around the median, indicating improved data consistency. Principal component analysis was further used to assess the effectiveness of batch effect removal (Fig. 3B). Before correction, samples clustered according to their respective batches; after correction, samples from different datasets overlapped closely in the principal component space, demonstrating that batch effects were effectively mitigated and systematic variation between datasets was minimized. Subsequently, differential expression analysis was performed on the training cohort. Based on the criteria, a total of 541 DEGs were identified, including 320 upregulated genes and 221 downregulated genes in OA samples (Table S3, Supplemental Digital Content, https://links.lww.com/MD/Q594). The results are presented as a volcano plot (Fig. 3C) and heatmap (Fig. 3D).

#### 3.2.2. WGCNA combined with differential expression analysis to identify OA key genes

**Figure 4.**
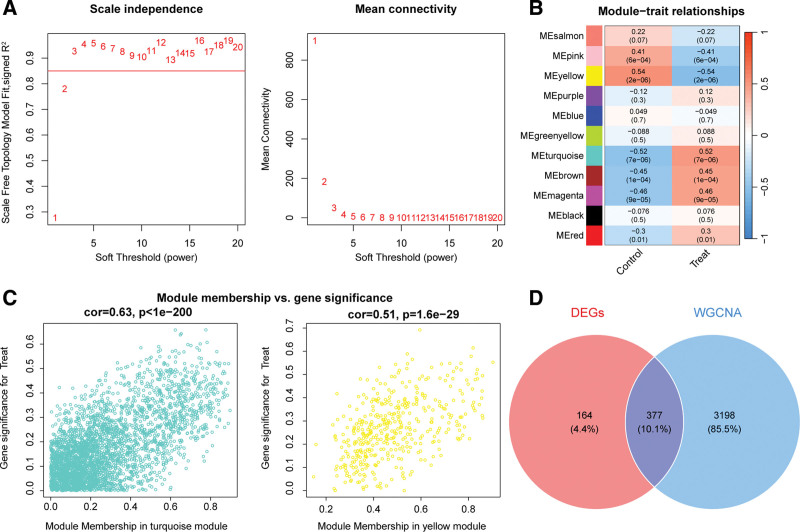
Identification of OA key genes by integrating WGCNA and differential expression analysis. (A) Determination of the optimal soft-threshold power using the dynamic tree cut algorithm; (B) Heatmap showing correlations between gene modules and OA phenotype; (C) scatter plots of module membership versus gene significance for the 2 key modules; (D) Venn diagram showing the union of DEGs and WGCNA-identified genes. DEGs = differentially expressed genes, OA = osteoarthritis, WGCNA = weighted gene co-expression network analysis.

WGCNA was performed on the training cohort to identify gene modules significantly associated with OA. As shown in Figure 4A, a soft-threshold power of 3 was selected, where the scale-free topology fit index (*R*^2^) first exceeded 0.85, indicating the network conformed to scale-free topology.Using a minimum module size of 80 genes and merging similar modules, 11 co-expression modules were identified (Fig. 4B). Modules were color-coded, and their module eigengenes were correlated with the OA phenotype using Pearson correlation. The yellow module showed a significant negative correlation with OA (*r* = −0.54, *P* = 2 × 10^−6^), comprising 425 genes; the turquoise module was positively correlated with OA (*R* = 0.52, *P* = 7 × 10^−6^), containing 3150 genes (Fig. 4B). Module significance analysis further confirmed strong correlations between the turquoise (*R* = 0.63, *P* < .05) and yellow (*R* = 0.51, *P* < .05) modules and OA (Fig. 4C). Accordingly, genes from these 2 modules were combined as WGCNA-derived OA susceptibility genes (Table S4, Supplemental Digital Content, https://links.lww.com/MD/Q594). Merging these genes with the previously identified DEGs resulted in a total of 3739 OA key genes (Fig. 4D).

### 3.3. Identification of BPA-related disease targets and enrichment analysis in OA pathogenesis

**Figure 5.**
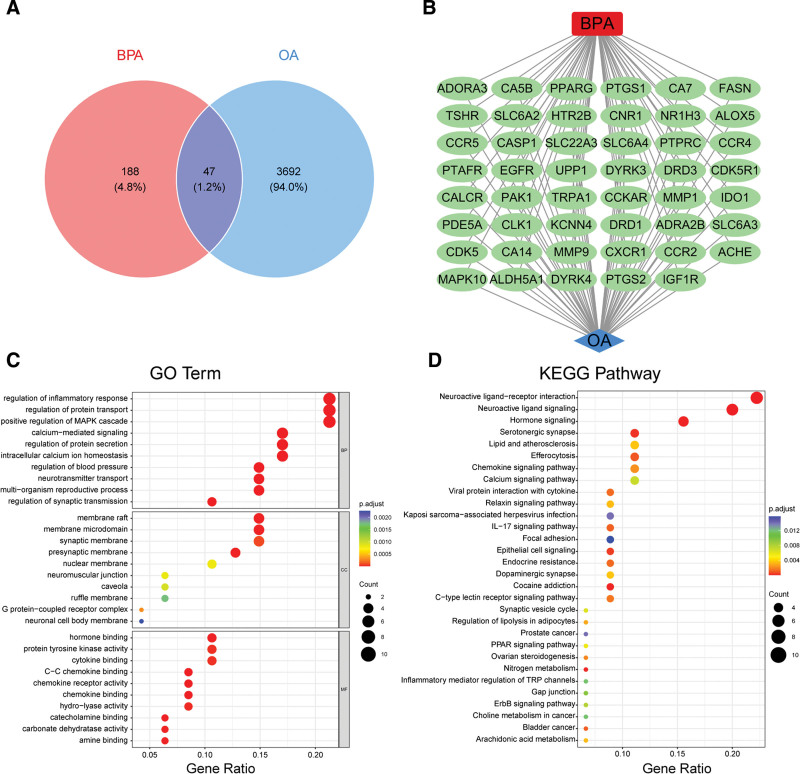
Intersection of BPA and OA key genes and functional enrichment analysis. (A) Venn diagram showing the overlap between predicted BPA targets and OA key genes; (B) regulatory network of overlapping BPA–OA genes; (C) GO functional enrichment analysis of intersecting genes; (D) KEGG pathway enrichment analysis of intersecting genes. BPA = Bisphenol A, GO = Gene Ontology, KEGG = Kyoto Encyclopedia of Genes and Genomes, OA = osteoarthritis.

To investigate potential targets through which BPA may influence OA pathogenesis, the predicted 235 BPA targets were intersected with the 3739 identified OA key genes, resulting in 47 overlapping genes (Fig. 5A). A BPA–OA target regulatory network was constructed to visualize potential regulatory relationships, highlighting genes such as PPARG, PTGS1, EGFR, MMP9, CCR5, CNR1, and DYRK4 as possible mediators of BPA’s effects on OA (Fig. 5B).

Gene Ontology (Table S5, Supplemental Digital Content, https://links.lww.com/MD/Q594) annotation revealed that these intersecting genes were significantly enriched in biological processes including regulation of inflammatory response, positive regulation of MAPK signaling pathway, calcium ion signaling, synaptic transmission, and neurotransmitter transport (Fig. 5C). These genes were predominantly localized to synaptic membranes, membrane rafts, and presynaptic neuron membranes, and were involved in molecular functions such as G protein-coupled receptor activity, chemokine activity, and enzyme binding. Kyoto Encyclopedia of Genes and Genomes pathway analysis (Table S6, Supplemental Digital Content, https://links.lww.com/MD/Q594) indicated significant enrichment of these 47 genes in multiple pathways closely related to OA and inflammation, including neuroactive ligand–receptor interaction, cytokine signaling pathways, calcium signaling, lipid metabolism, TRP channels, and PPAR signaling pathways (Fig. 5D). These findings suggest that BPA may modulate OA pathogenesis by interfering with inflammation, metabolic, and neuroregulatory signaling axes.

### 3.4. Multi-model prediction and SHAP feature interpretation identify core genes

**Figure 6.**
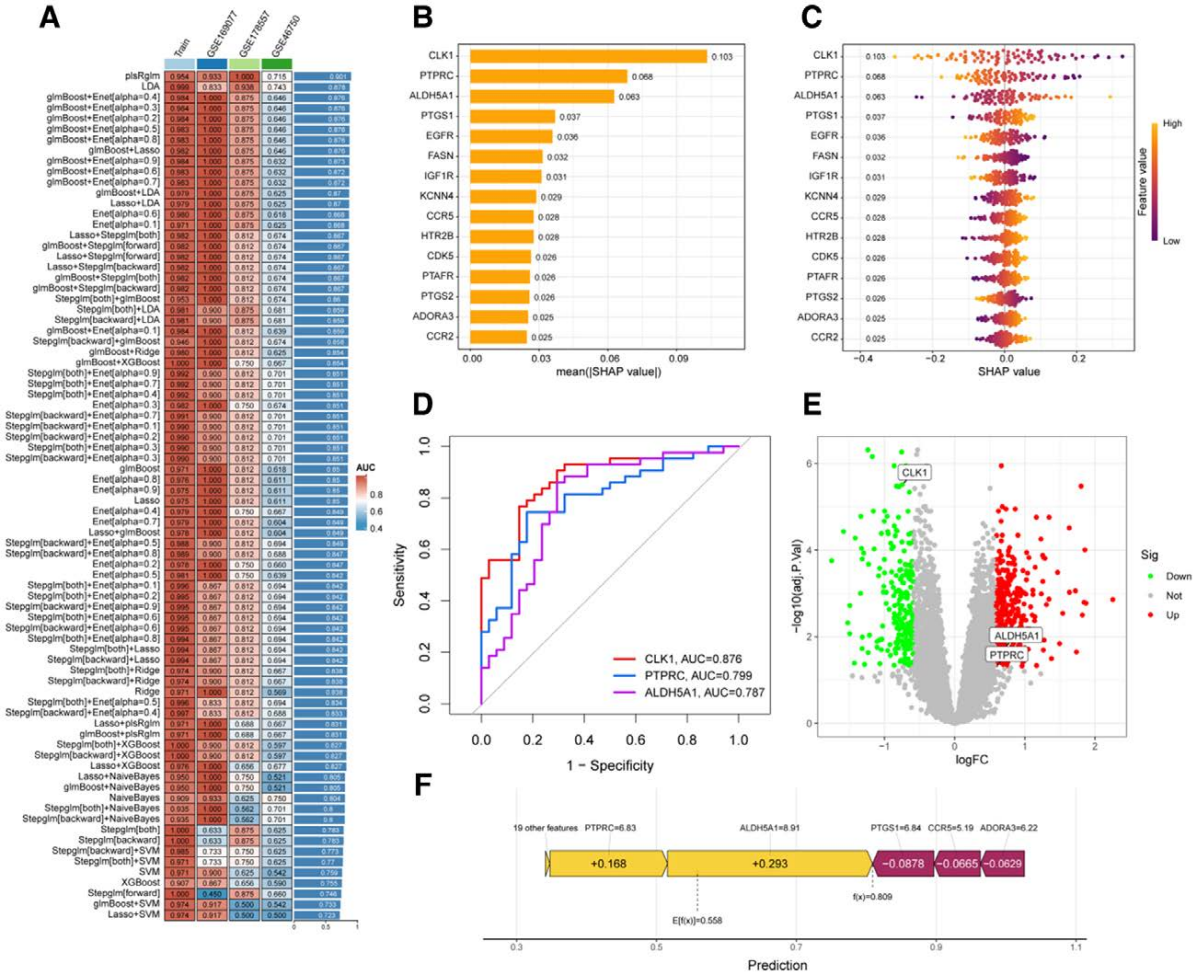
Identification of core genes induced by BPA in OA. (A) Heatmap comparing model performance by average AUC values across cohorts; (B) Bar plot ranking feature importance of genes; (C) Violin plots showing gene expression distributions under different conditions; (D) receiver operating characteristic curves for the 3 core genes; (E) Volcano plot illustrating expression changes of core genes; (F) SHAP summary plot displaying contribution of feature genes to model predictions. AUC = area under the receiver operating characteristic curve, BPA = Bisphenol A, OA = osteoarthritis, SHAP = SHapley Additive exPlanations.

Comprehensive machine learning analysis was performed on the 47 overlapping targets by constructing 113 predictive models to identify core genes through which BPA may influence OA. The plsRglm ensemble model demonstrated the best generalization performance in both training and testing cohorts, achieving an average AUC of 0.901, outperforming other models (Fig. 6A), and was thus selected as the optimal model. SHAP were applied to interpret the contribution of each feature gene in the optimal model. As shown in Figure 6B, CLK1 (SHAP = 0.103), PTPRC (SHAP = 0.068), and ALDH5A1 (SHAP = 0.063) had the highest importance scores and were identified as the most influential predictors. CLK1 exhibited a negative regulatory effect on OA, whereas PTPRC and ALDH5A1 showed positive regulatory effects (Fig. 6C). Receiver operating characteristic curve analysis confirmed the diagnostic potential of these core genes with AUCs > 0.7 (Fig. 6D). Their differential expression patterns in OA samples were visualized using a volcano plot (Fig. 6E). Furthermore, SHAP force plots (Fig. 6F) indicated that PTPRC (value = 6.83, Δ = 0.168) and ALDH5A1 (value = 8.91, Δ = 0.293) were major drivers increasing the predicted model output, resulting in a final prediction value *f*(*x*) = 0.809, substantially above the model expectation *E*[*f*(*x*)] = 0.55. In summary, this integrative strategy combining ensemble machine learning and explainable modeling successfully identified CLK1, PTPRC, and ALDH5A1 as potential core regulators mediating BPA’s involvement in OA pathogenesis.

### 3.5. Molecular docking validation of BPA interaction with core targets

**Figure 7.**
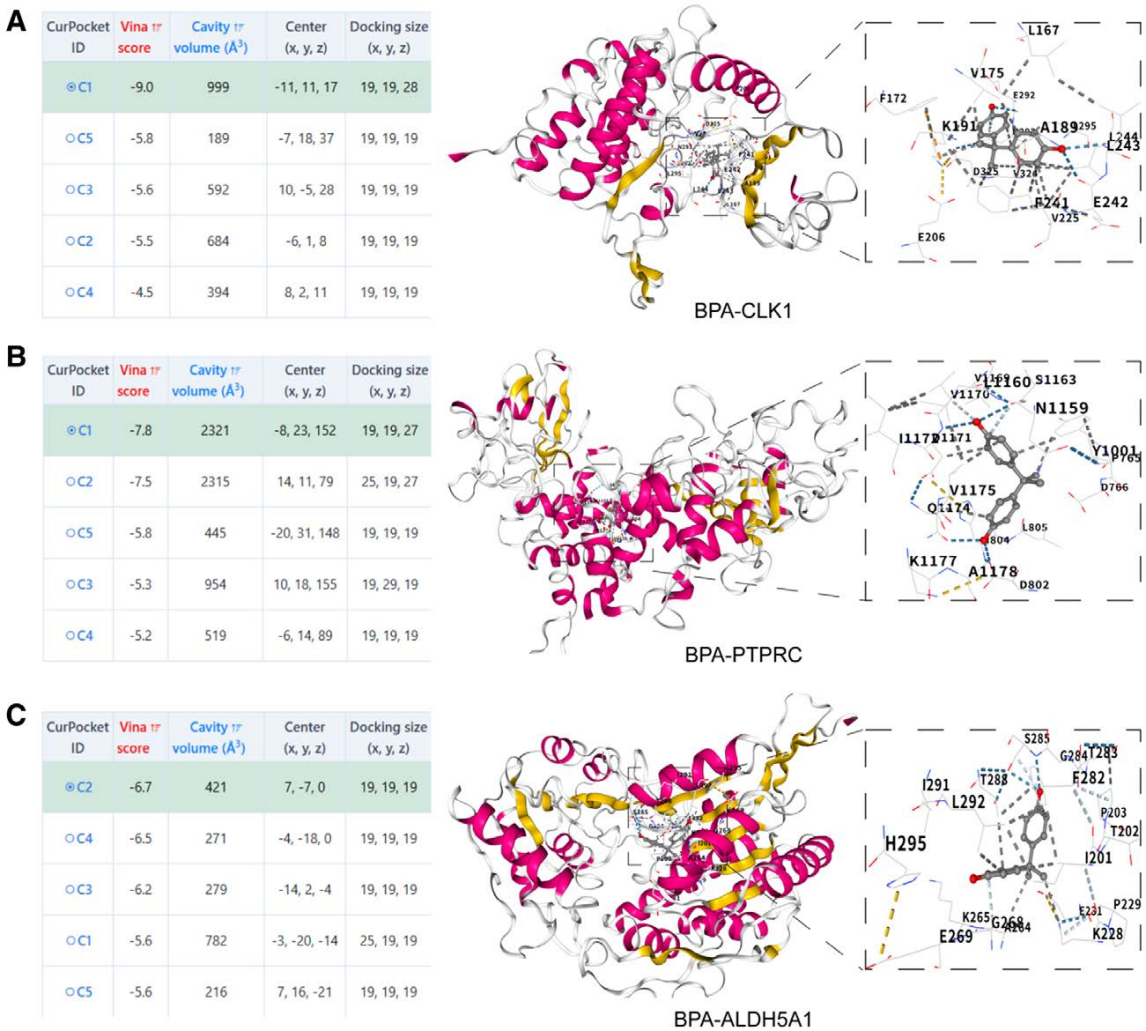
Molecular docking validation of interactions between BPA and core genes. (A) Docking results and visualization of BPA with CLK1; (B) docking results and visualization of BPA with PTPRC; (C) docking results and visualization of BPA with ALDH5A1. BPA = Bisphenol A.

To validate the potential binding interactions between BPA and the identified core genes (CLK1, PTPRC, and ALDH5A1), molecular docking was performed, with 5 binding poses generated per docking. The results demonstrated strong binding affinities between BPA and these 3 target proteins, with the lowest binding energies consistently below −5 kcal/mol, indicating stable and spontaneous molecular interactions (Fig. 7).

According to established criteria in molecular docking studies, binding energies <0 kcal/mol indicate spontaneous binding, while energies below −5.0 kcal/mol suggest excellent binding affinity. Visualization of the docking conformations (Fig. 7) revealed the most stable binding modes for all BPA–protein complexes. These findings provide molecular evidence supporting direct interactions between BPA and the OA core targets identified via our machine learning approach.

## 4. Discussion

In recent years, increasing evidence has highlighted the important role of environmental exposure factors in the pathogenesis and progression of OA, with particular attention to endocrine-disrupting chemicals regulating joint tissue homeostasis and inflammatory responses.^[21,22]^ BPA, a prevalent environmental chemical, has been shown to induce inflammation, oxidative stress, and chondrocyte damage: processes closely aligned with the pathological features of OA.^[23,24]^ Despite extensive research on BPA’s toxicological effects in metabolic disorders, endocrine disruption, and immune dysregulation, its precise molecular mechanisms and key targets in OA remain unclear. This knowledge gap limits our understanding of the environmental etiology of OA and poses challenges for environmental intervention and risk assessment. Therefore, systematically elucidating the molecular links and potential regulatory mechanisms between BPA and OA is of great significance for revealing the environmental pathogenesis of OA and developing prevention strategies.

By integrating multi-omics datasets with advanced machine learning and bioinformatics approaches, we systematically identified potential molecular targets connecting BPA exposure to OA pathogenesis. Our network toxicology and bioinformatics analyses revealed 47 candidate genes, among which 3 core genes (CLK1, PTPRC, and ALDH5A1) stood out. Differential expression analysis showed downregulation of CLK1 and upregulation of PTPRC and ALDH5A1 in OA samples. Machine learning models confirmed the high diagnostic value of these gene features, with SHAP interpretability analysis emphasizing CLK1 (SHAP value = 0.103) as the most influential predictor. Molecular docking simulations further supported the biological relevance of these findings, revealing strong binding affinities between BPA and the core genes mediated by specific amino acid interactions. Collectively, these results suggest that the identified core genes may represent key molecular targets through which BPA exerts its effects in OA pathogenesis.

CLK1 is a dual-specificity protein kinase capable of phosphorylating serine, threonine, and tyrosine residues. It is primarily localized in the nucleus and plays a critical role in regulating phosphorylation of SR proteins and pre-mRNA splicing processes.^[25]^ Studies have demonstrated that CLK1 phosphorylates key splicing factors such as U1-70K and SRSF1, altering their conformation and promoting the assembly of U1 small nuclear ribonucleoprotein, thereby regulating splice site recognition.^[26,27]^ Aberrant RNA splicing is closely associated with chronic inflammation and various degenerative diseases. In OA, splicing dysregulation may lead to abnormal expression of matrix-degrading enzymes such as MMPs and ADAMTS, disrupting cartilage homeostasis.^[28–30]^ Moreover, environmental toxicants like BPA can interfere with RNA splicing by affecting epigenetic regulation and post-transcriptional mechanisms, including inducing alternative splicing events and modulating spliceosome expression.^[31,32]^ These findings suggest that CLK1 may serve as a key node through which BPA dysregulates RNA splicing, thereby indirectly contributing to OA pathogenesis.

PTPRC, also known as CD45, is a protein tyrosine phosphatase expressed in all nucleated hematopoietic cells, including T cells, B cells, and macrophages, and plays a central role in regulating immune cell activation and signal transduction.^[33,34]^ Studies have shown that in traumatic OA synovial tissue, PTPRC gene expression is highly enriched in immune cell populations, particularly dominating monocyte-macrophage, T cell, and dendritic cell subsets.^[35]^ Proteomic analyses have also detected PTPRC expression in OA synovium, with its expression levels correlating to inflammatory status; although more prominent in rheumatoid arthritis, this suggests a potential role of PTPRC in the immune microenvironment of OA synovium.^[36]^ Recent focused studies observed upregulation of PTPRC in both OA cartilage and synovial tissues, showing positive correlations with matrix degradation, cell death, and inflammatory cytokine expression (e.g., PTPRC alongside MAPK14 and PTPN12 has been identified as a frequently differentially expressed gene in OA).^[37]^ As a key immune cell marker, PTPRC may promote synovial inflammation and cartilage degradation by modulating immune cell infiltration in the synovium and the production of pro-inflammatory mediators such as IL-1β and TNF-α.^[35]^ Thus, the identification of PTPRC as a core BPA–OA target in our study is biologically plausible. BPA may influence the functional status of synovial and peripheral immune cells by regulating the PTPRC-mediated immune regulatory network, contributing to OA pathogenesis and progression within a low-grade inflammatory microenvironment.

ALDH5A1 encodes a mitochondrial NAD⁺-dependent succinic semialdehyde dehydrogenase, a key enzyme in the γ-aminobutyric acid (GABA) metabolic pathway that converts succinic semialdehyde to succinate, thus participating in energy metabolism and antioxidant homeostasis regulation.^[38]^ Deficiency of this enzyme leads to disrupted GABA metabolism and accumulation of oxidative metabolic intermediates, resulting in neurological impairments in hereditary metabolic disorders.^[38,39]^ Recent cellular and molecular studies on OA have demonstrated that oxidative stress and mitochondrial dysfunction play central roles in OA pathogenesis. Accumulation of reactive oxygen species induces chondrocyte apoptosis, extracellular matrix degradation, and dysregulation of signaling pathways such as NF-κB and MAPK.^[40–42]^ ALDH5A1 exerts a detoxifying function in antioxidant responses; its downregulation may exacerbate reactive oxygen species accumulation and mitochondrial damage, thereby disrupting cartilage metabolic homeostasis. Our findings suggest that BPA, known to induce oxidative stress and inhibit antioxidant enzyme activities, may further impair ALDH5A1 function, aggravating oxidative damage and promoting cartilage degeneration in OA. Additionally, this gene may link GABA metabolism with regulation of the joint microenvironment, offering a novel perspective on the metabolic–inflammation–cartilage homeostasis axis. The synergistic roles of these genes highlight the complex network of inflammation, metabolic dysregulation, and oxidative stress, providing a theoretical basis and key targets for future mechanistic studies and targeted interventions.

Our findings also carry important implications for public health and policy. The identification of BPA as a potential contributor to OA development highlights the necessity of stricter regulation of environmental endocrine disruptors. Reducing BPA exposure through improved industrial standards, safer alternatives in consumer products, and strengthened environmental monitoring could help mitigate the risk of environmentally driven OA. Moreover, raising public awareness about minimizing daily contact with BPA-containing materials may serve as an effective preventive measure. Together, these considerations emphasize the value of translating molecular toxicology insights into practical strategies for environmental health protection.

Inevitable limitations exist in this study. First, although the sample size meets the basic requirements for exploratory analysis, it may still limit the external generalizability of the results, thereby affecting their applicability to larger populations. Second, despite partial control of confounding factors such as age, genetic background, and lifestyle during analysis, residual confounding effects cannot be completely excluded and may influence the findings to some extent. Third, the predictive results of this study are primarily based on bioinformatics and computational modeling and require further validation of their biological relevance and accuracy through in vitro and in vivo experiments. Future research should expand cohort sizes and increase sample diversity, particularly focusing on populations with high BPA exposure, to better elucidate the mechanisms in specific susceptible groups. Moreover, integrating molecular biology experiments, animal models, and clinical follow-up from multiple dimensions will help further verify and refine the findings at a mechanistic level, enhancing the reliability and translational value of the conclusions. Nevertheless, this study provides novel evidence and insights into the mechanisms by which BPA may mediate OA pathogenesis, carrying important implications for the prevention and control of environmentally related osteoarticular diseases and for public health policy formulation.

## 5. Conclusion

This study identified 47 overlapping genes between BPA targets and OA-related genes, with CLK1, PTPRC, and ALDH5A1 highlighted as core targets. Molecular docking confirmed stable binding of BPA to these proteins, providing novel molecular evidence for the environmental contribution to OA. These findings suggest that BPA exposure may be a modifiable risk factor for OA and highlight the need for future studies on dose–response relationships and preventive interventions.

## Acknowledgments

We gratefully acknowledge the authors of the GEO datasets used in this study for their valuable contributions and for making their data publicly accessible.

## Author contributions

**Data curation:** Jing Tan.

**Formal analysis:** Jing Tan.

**Funding acquisition:** Daobin Han.

**Methodology:** Jing Tan, Daobin Han.

**Software:** Jing Tan.

**Supervision:** Jing Tan, Daobin Han.

**Validation:** Jing Tan, Daobin Han.

**Writing – original draft:** Jing Tan.

**Writing – review & editing:** Daobin Han.

## Supplementary Material




